# Transactions of the Virginia Society of Surgeon Dentists

**Published:** 1843-12

**Authors:** 


### ARTICLE V.

*Transactions of the Virginia Society of Surgeon Dentists*, at their Annual Meeting, held in the office of Dr. Sam'l Lethbridge, in the City of Richmond, October 11th, 1843.

At 11 o'clock, A. M., the President, Dr. S. Lethbridge, took the chair. The Secretary called the roll, and read the excuses of absentees. The proceedings of the last meeting were read.

A vacancy having occurred in the Examining Committee, in consequence of the absence of some of the members, on motion, the said vacancy was filled by the appointment of Drs. John G. Wayt, S. M. Sheppard and J. W. Sheppard.

The Examining Committee reported to the Society the name of Solomon Angle, who had been rigidly examined, on the theory and practice of Dental Surgery, and who they recommend to membership in the Society, and to the degree of D. D. S.

### *Afternoon Session.*

The committee appointed to address Dr. R. N. Hudson, asking explanation, in relation to certain opprobrious remarks, involving character before the Society, made the following report:

"The committee appointed by the Virginia Society of Surgeon Dentists, to investigate certain charges alleged against Dr. R. N. Hudson, a member of said Society, beg leave to *report*, that having examined the testimony upon which these charges were founded, and politely and respectfully offered him an opportunity of vindicating himself, which he most *rudely* and abruptly declined; thereby, in the opinion of the committee, *tacitly* acknowledging the truth of the allegations in question; in consideration of which fact, in connection with the character of the evidence adduced, the committee beg leave to suggest the expulsion of Dr. R. N. Hudson from membership in said Society.

Signed, W. W. H. THACKSTON, Chairman.

*Wednesday, Oct. 11th,* 1843."

Whereupon, it was

*Resolved,* That the name of Dr. R. N. Hudson be stricken from the roll of members for contumacy.

*Evening Session.*

The Society assembled pursuant to adjournment at 4 o'clock, P. M.

The President called to order. The Treasurer presented his report, which was approved and ordered to be filed with the papers of the Society.

On motion of Dr. S. M. Sheppard, the Society proceeded to the election of officers for the ensuing year, when the following gentlemen were declared duly elected:

Dr. SAMUEL LETHBRIDGE, *President.*
" JOHN G. WAYT, *Vice-president.*
" JAMES D. McCABE, *Cor. and Rec. Secretary.*
" S. M. SHEPPARD, *Treasurer.*
" W. W. H. THACKSTON,
" JOHN McCONNELL,
" S. B. HAMBLIN,
" JOHN W. SHEPPARD,
" WM. N. McKENNEY,
} Executive, Examining and Publishing Committee.

On motion, Dr. W. W. H. Thackston was elected, and requested to deliver the Annual Address, at the next Annual Meeting.

The following gentlemen were appointed to prepare Essays to be read at the next Annual Meeting of the Society, it being left to themselves to select subjects. Drs. J. G. Wayt, J. McConnell, J. M. Sheppard, John C. McCabe, S. Angle, S. Lethbridge, Jas. D. McCabe, and W. C. Crump.

On motion of Dr. James D. McCabe, the following resolution was adopted:

*Resolved,* That this Society, as a token of their high esteem for the distinguished professional labors and personal merit of Prof. C. A. Harris of the Baltimore College of Dental Surgeons, create him an Honorary Member of their body; and that he be furnished with the Society's diploma, without the usual fee.

On motion of Dr. W. W. H. Thackston,

*Resolved,* That as an evidence of the esteem of this Society for the professional and personal merits of Prof. H. H. Hayden of the Baltimore College of Dental Surgeons, create him an Honorary Member of their body, and present him with the diploma of the Society, without the usual fee.

The Annual Address was then pronounced before the Society, by Dr. James D. McCabe.

Dr. W. W. H. Thackston read an Essay on the "Morbid Effects of Diseased Teeth upon the Human System."

On motion,

*Resolved,* That Dr. McCabe be requested to furnish a copy of the Annual Address, delivered before the Society this evening, for publication in the American Journal of Dental Science.

On motion,

*Resolved,* That Dr. W. W. H. Thackston be requested to furnish a copy of his Essay, read before the Society this evening, for publication in the American Journal of Dental Science.

On motion,

*Resolved,* That the thanks of the Society are due to the President, Vice-president, Secretary, and Treasurer, for the able manner in which their duty has been discharged.

On motion,

*Resolved,* That the thanks of the Society be presented to the Examining Committee, for the able firm and intelligent manner with which they have conducted the examinations at this meeting.

On motion,

*Resolved,* That the committee appointed at the formation of this Society, to procure a seal, be, and are hereby instructed to procure and superintend the engraving and printing of a copperplate diploma, for the use of the Society.

On motion,

*Ordered,* That the next Annual Meeting of the Society be held in the town of Petersburg.

On motion,

*Ordered,* That this Society do now adjourn to the second Wednesday in October, 1844.

S. LETHBRIDGE, D. D. S., President.

James D. McCabe, D. D. S., Cor. and Rec. Sec'y.

